# O-GlcNAcylation enhances sensitivity to RSL3-induced ferroptosis via the YAP/TFRC pathway in liver cancer

**DOI:** 10.1038/s41420-021-00468-2

**Published:** 2021-04-16

**Authors:** Guoqing Zhu, Abduh Murshed, Haojie Li, Ji Ma, Ni Zhen, Miao Ding, Jiabei Zhu, Siwei Mao, Xiaochen Tang, Li Liu, Fenyong Sun, Lei Jin, Qiuhui Pan

**Affiliations:** 1 Department of Clinical Laboratory Medicine, Shanghai Children’s Medical Center, School of Medicine, Shanghai Jiaotong University, 200127 Shanghai, China; 2 Department of Clinical Laboratory, Shanghai Tenth People’s Hospital of Tongji University, 200072 Shanghai, China; 3 Department of Clinical Laboratory, Shanghai Fourth People’s Hospital Affiliated to Tongji University School of Medicine, 200434 Shanghai, China; 4 Faculty of Medical Laboratory, Shanghai University of Medicine and Health Sciences, 201318 Shanghai, China

**Keywords:** Cell death, Cancer

## Abstract

Ferroptosis is a form of regulated cell death characterized by iron-dependent accumulation of lipid hydroperoxides to lethal levels. YAP has been reported to play a pivotal role in controlling ferroptotic death, and the expression of YAP is enhanced and stabilized by O-GlcNAcylation. However, whether O-GlcNAcylation can increase the sensitivity of hepatocellular carcinoma (HCC) cells to ferroptosis remains unknown. In the present study, we found that O-GlcNAcylation increased the sensitivity of HCC cells to ferroptosis via YAP. Moreover, YAP increased the iron concentration in HCC cells through transcriptional elevation of TFRC via its O-GlcNAcylation. With YAP knockdown or YAP-T241 mutation, the increased sensitivity to ferroptosis induced by O-GlcNAcylation was abolished. In addition, the xenograft assay confirmed that O-GlcNAcylation increased ferroptosis sensitivity via TFRC in vivo. In summary, we are the first to find that O-GlcNAcylation can increase ferroptosis sensitivity in HCC cells via YAP/TFRC. Our work will provide a new basis for clinical therapeutic strategies for HCC patients.

## Introduction

Ferroptosis is a newly identified form of programmed cell death that is different from apoptosis and necrosis^1^, and characterized by iron-dependent accumulation of lipid peroxides^2,3^. Ferroptosis is mostly induced by the small molecule erastin, which blocks the cysteine-glutamate transporter (system Xc^−^) to reduce glutathione (GSH) production^1,4^ but can also be induced by RSL3, which directly inhibits the activity of glutathione peroxidase 4 (GPX4), leading to lipid-reactive oxygen species (ROS) accumulation^5,6^. In addition, multiple genes and pathways have been found to regulate ferroptosis by modulating iron metabolism, amino acid metabolism, and lipid metabolism. For example, ACSL4 is responsible for the synthesis of polyunsaturated fatty acid-PEs (PUFA-PEs), and a decrease in ACSL4 leads to ferroptosis resistance^7^. Importantly, iron metabolism, which is required for lipid ROS accumulation by the Fenton reaction, is essential for ferroptosis initiation^8,9^. Thus, an elevated iron concentration has an obvious impact on ferroptosis sensitivity. The transferrin receptor (TFRC) and transferrin (TF), which mediate iron import, are required for the promotion of ferroptosis sensitivity^10,11^. Previous studies reported that knockdown of TFRC suppresses erastin-induced ferroptosis^12^. Gao et al. also indicated that inhibition of TFRC suppresses ferroptosis induced by amino acid/cysteine deprivation^13^. The biological function of ferroptosis under normal physiological conditions remains unexplained. Evidence has indicated that ferroptosis might be a breakthrough therapy for cancer by regulating critical nutrients^14^. Explorations focusing on how to effectively kill cancer cells while leaving normal cells unharmed have led to the identification of ferroptosis as a promising alternative method.

O-GlcNAcylation is a reversible posttranslational modification catalyzed by O-GlcNAc transferase (OGT)^15^. Generally, the UDP-GlcNAc donor is transferred to serine and/or threonine residues in substrate proteins to regulate the expression, stability, subcellular location, and function of these proteins^16^. O-GlcNAcylation often functions as a nutrient sensor to modify multiple aspects of cell physiology, especially in cancer^17^. Cancer cells take up large amounts of nutrients and preferentially use aerobic glycolysis, which greatly increases global O-GlcNAcylation, to adapt to the rapid growth of tumors. Numerous studies have confirmed increased O-GlcNAcylation in various tumor tissues compared to adjacent normal tissues, for example, in breast cancer, lung cancer, and colon cancer^18^. In our previous study, we observed elevated O-GlcNAcylation in liver cancer and found that overexpression of OGT facilitated hepatocellular carcinoma (HCC) tumorigenesis^19^. Importantly, we first found that OGT can modify threonine (Thr) 241 O-GlcNAcylation of yes-associated protein (YAP), which is the core factor in the Hippo pathway, to facilitate its transcriptional activity. However, O-GlcNAcylated YAP, in turn, accelerates glucose metabolism by activating the hexosamine biosynthetic pathway (HBP). YAP and O-GlcNAcylation form a harmful axis to promote glucose and lipid metabolism in order to facilitate tumorigenesis.

Intriguingly, a recent study published in *Nature* made an important discovery that highly confluent cells exhibited increased resistance to ferroptosis and that this ferroptosis resistance relied on YAP expression^20^. Elevated YAP phosphorylation and cytoplasmic localization mitigated the ferroptosis sensitivity of cancer cells. Moreover, the function of the YAP protein is dependent on O-GlcNAcylation.

From a metabolic perspective, increasing glucose import into cancer cells promotes metabolic reprogramming by contributing to glycolysis and the HBP pathway, the metabolic branch pathway of glycolysis^21^. Ferroptosis is closely associated with nutrient metabolism, including amino acid metabolism and lipid metabolism. Since O-GlcNAcylation and ferroptosis are both metabolic physiological processes, we sought to explore the potential relationship between O-GlcNAcylation and ferroptosis. In the present study, we found that O-GlcNAcylation can enhance sensitivity to RSL3-induced ferroptosis via the YAP/TFRC pathway. Our findings might provide a new approach for cancer therapy via ferroptosis induction.

## Results

### O-GlcNAcylation increases the sensitivity of HCC cells to ferroptosis

**Fig. 1:**
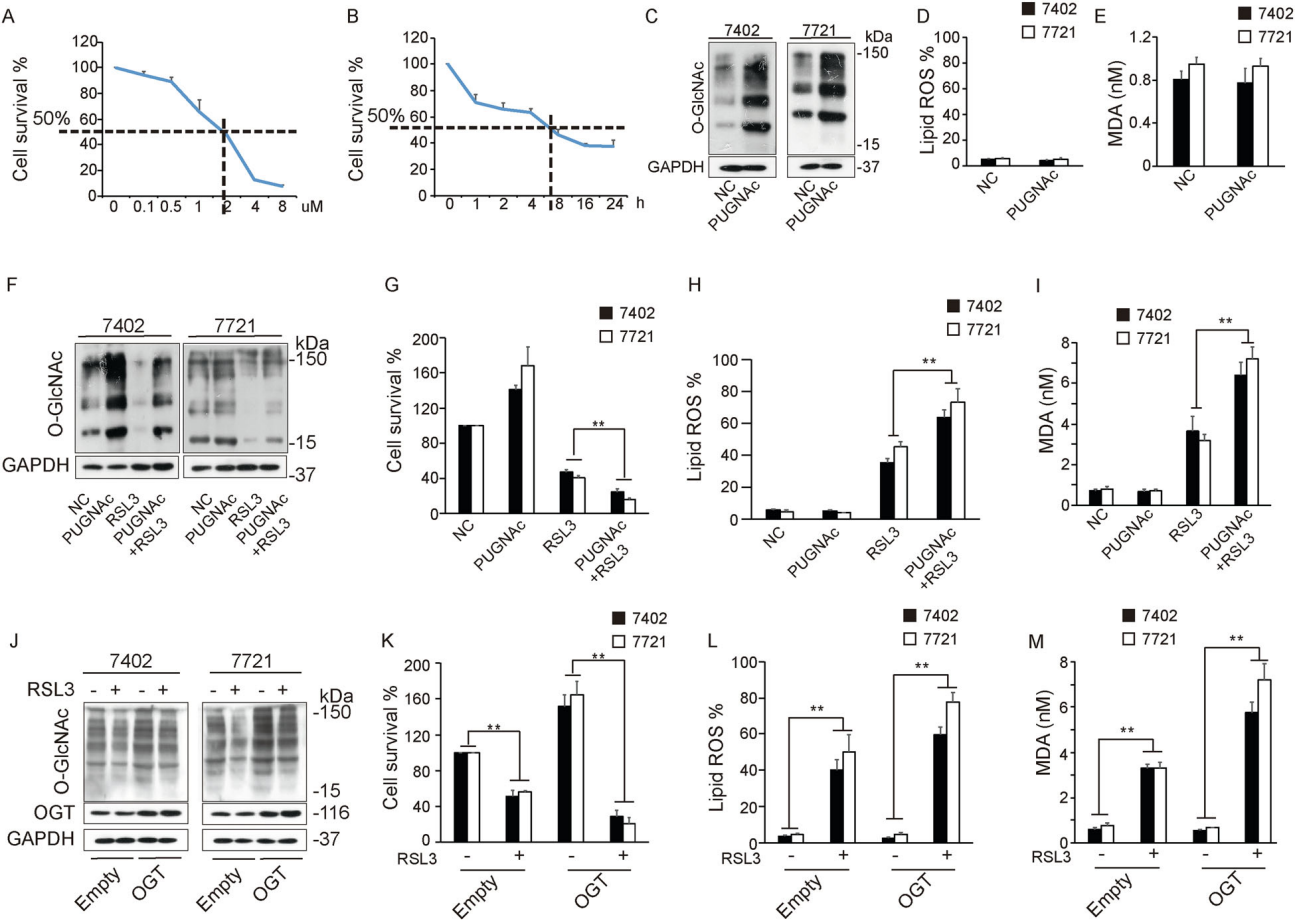
O-GlcNAcylation increases ferroptosis sensitivity of HCC cells. **A** and **B** Cell viability was measured by an MTT assay in SMMC-7721 cells treated with RSL3 at the indicated concentration (**A**) and for the indicated time (**B**). **C** Global O-GlcNAcylation was measured by WB after PUGNAc treatment (25â€‰µM). **D** The fluorescence of C11-BODIPY was measured to detect lipid ROS in Bel-7402 and SMMC-7721 cells treated as indicated. **E** MDA levels were measured in Bel-7402 and SMMC-7721 cells treated as indicated. **F** Global O-GlcNAcylation was detected by WB in cells treated with PUGNAc and RSL3 separately or simultaneously. **G**–**I** Ferroptotic events were evaluated in cells treated with PUGNAc and RSL3 separately or simultaneously: cell death (**G**), lipid ROS production (**H**), and MDA production (**I**). **J** Global O-GlcNAcylation and OGT expression were detected by WB in Bel-7402 and SMMC-7721 cells overexpressing OGT. Cells were subsequently treated with RSL3 as indicated. **K**–**M** Ferroptotic events were evaluated in cells overexpressing OGT and treated with RSL3 as indicated: cell death (**K**), lipid ROS production (**L**), and MDA production (**M**).

RSL3 is recognized as a classical small molecule that induces ferroptosis by inhibiting the PL-peroxidase activity of GPX4. To explore the suitable concentration and timing of drug intervention, we performed concentration gradient and time point assays. The results indicated that the cells should be treated with RSL3 at a concentration of 2 µM for ~6 h (Fig. 1A, B). To investigate the relationship between O-GlcNAcylation and ferroptosis, we induced O-GlcNAcylation by treatment with PuGNAc, an acknowledged O-GlcNAcylation inducer (Fig. 1C). We observed that PuGNAc barely influenced the level of lipid ROS generation, a specific marker of ferroptosis, as detected by C11-BODIPY staining (Fig. 1D). Assays of MDA, the major end product of lipid peroxidation, showed the same results (Fig. 1E). Since ferroptosis and O-GlcNAcylation are both closely related to metabolism, and high O-GlcNAcylation induction leads to an increase in overall cell metabolism, disruption of the balance may cause changes in ferroptosis sensitivity. We hypothesized that O-GlcNAcylation-induced high metabolism levels might change the sensitivity to ferroptosis. Thus, we induced ferroptosis with RSL3 after PUGNAc treatment (Fig. 1F). Surprisingly, we found by measuring cell viability that compared to RSL3 treatment alone, pretreatment with PuGNAc significantly enhanced RSL3-induced ferroptosis (Fig. 1G). In addition, as the major ferroptotic events, RSL3-induced lipid ROS generation and MDA production were significantly increased after PUGNAc treatment (Fig. 1H, I). Subsequently, we generated an OGT overexpression plasmid, which led to global O-GlcNAcylation upregulation (Fig. 1J). We explored the typical ferroptotic events. Similarly, the results indicated that overexpression of OGT enhanced RSL3-induced cell death (Fig. 1K), lipid ROS generation (Fig. 1L) and MDA production (Fig. 1M). Moreover, to confirm that O-GlcNAcylation contributes to sensitivity to ferroptosis rather than other forms of cell death, such as apoptosis or necroptosis, we treated cells with the ferroptosis inhibitor ferrostatin-1, the apoptosis inhibitor ZVAD-FMK and the necroptosis inhibitor necrosulfonamide. The results indicated that PUGNAc contributed to ferroptosis sensitivity and that its effects could be reversed by ferrostatin-1 only (Supplementary Fig. 1A, B), which confirmed our hypothesis. Importantly, we further knocked down OGT expression to inhibit global O-GlcNAcylation (Supplementary Fig. 1C). We found that RSL3-induced ferroptosis was significantly attenuated (Supplementary Fig. 1D–F). Together, these results indicated that O-GlcNAcylation increases the sensitivity of HCC cells to ferroptosis.

### O-GlcNAcylated YAP mediates the ferroptosis sensitivity of HCC cells

**Fig. 2:**
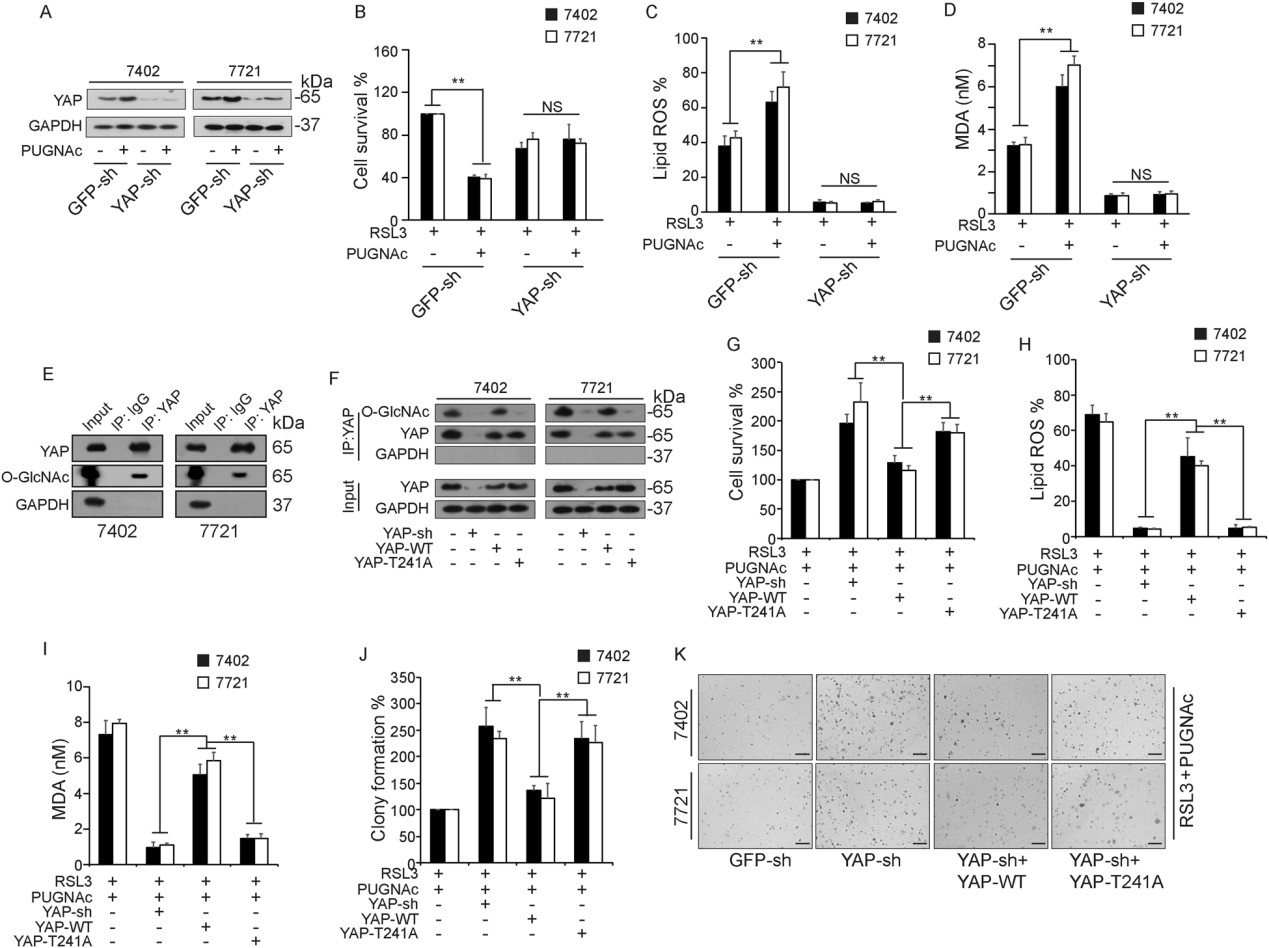
YAP O-GlcNAcylation mediates the ferroptosis sensitivity of HCC cells. **A** YAP expression levels were determined in control and YAP knockdown Bel-7402 and SMMC-7721 cells treated with PUGNAc. **B**–**D** After RSL3 pretreatment, ferroptotic events were evaluated in control and YAP knockdown Bel-7402 and SMMC-7721 cells treated with PUGNAc: cell death (**B**), lipid ROS production (**C**), and MDA production (**D**). **E** Interaction between O-GlcNAc and YAP using anti-YAP antibody with input as control. **F**–**I** Bel-7402 and SMMC-7721 cells were transfected with YAP-sh, and YAP-WT or YAP-T241A was simultaneously overexpressed. Endogenous YAP was immunoprecipitated, and O-GlcNAcylation of YAP was detected by WB (**F**). After RSL3 and PUGNAc pretreatment, ferroptotic events were evaluated in control and transfected Bel-7402 and SMMC-7721 cells: cell death (**G**), lipid ROS production (**H**), and MDA production (**I**). **J**, **K** After RSL3 and PUGNAc pretreatment, the colony formation capacity was measured using a soft agar assay in control cells, and Bel-7402 and SMMC-7721 cells transfected with YAP-sh with simultaneous overexpression of YAP-WT or YAP-T241A.

Our previous work demonstrated that high glucose levels can promote YAP O-GlcNAcylation and stimulate YAP activity, as also shown by Peng et al.^22^. Intriguingly, another study published in *Nature* by Wu et al. indicated that the transcriptional activity of YAP promotes ferroptosis^20^. This finding reminded us that YAP might mediate O-GlcNAcylation-enhanced ferroptosis. To validate our hypothesis, we knocked down YAP expression using a YAP lentiviral plasmid and then treated the cells with PUGNAc. Decreased YAP expression was confirmed using WB (Fig. 2A). MTT assays were conducted to measure the viability of Bel-7402 and 7404 cells that were pre-exposed to RSL3. The results indicated that PUGNAc treatment aggravated ferroptosis and that this phenomenon was abolished when YAP was knocked down (Fig. 2B). The levels of lipid ROS and MDA production were elevated after PUGNAc treatment, and these increases were reversed when YAP was knocked down (Fig. 2C, D). We previously demonstrated that YAP transcriptional activity relies on its at Thr241 O-GlcNAcylation^19^. By co-IP experiments using anti-YAP antibodies in both 7402 and 7721 cells, we confirmed that O-GlcNAc was detected in the immunoprecipitates pulled down (Fig. 2E). Moreover, we also detected YAP in the immunoprecipitates pulled down by using anti-O-GlcNAc antibodies in both 7402 and 7721 cells (Supplementary Fig. 2A). Enzymatic labeling of O-GlcNAc sites using HRP-labeled Streptavidin and anti-TAMRA antibodies directly confirmed that YAP can be O-GlcNAcylated (Supplementary Fig. 2B). Further, enzymatic labeling of O-GlcNAc sites also confirmed that YAP Thr241 was the major O-GlcNAcylation site of YAP (Supplementary Fig. 2C). To further investigate whether the O-GlcNAcylation of YAP Thr241 mediates the high glucose-induced enhancement of ferroptosis sensitivity, we simultaneously expressed YAP-T241A or wild type (WT) YAP in cells with downregulated YAP expression (Fig. 2F). As the results indicated, the increase in cell viability caused by YAP knockdown was significantly attenuated by overexpression of YAP-WT but not YAP-T241A in cells pretreated with PUGNAc and RSL3 (Fig. 2G). Similarly, the decreases in the levels of lipid ROS and MDA caused by YAP knockdown were also rescued by overexpression of YAP-WT but not YAP-T241A (Fig. 2H, I). Moreover, we performed colony formation assays to verify cell viability under the indicated treatment, and the results were consistent with those of the MTT assays (Fig. 2J, K). These results indicated that high glucose levels can enhance ferroptosis sensitivity via YAP O-GlcNAcylation.

### YAP regulates iron metabolism by targeting TFRC

**Fig. 3:**
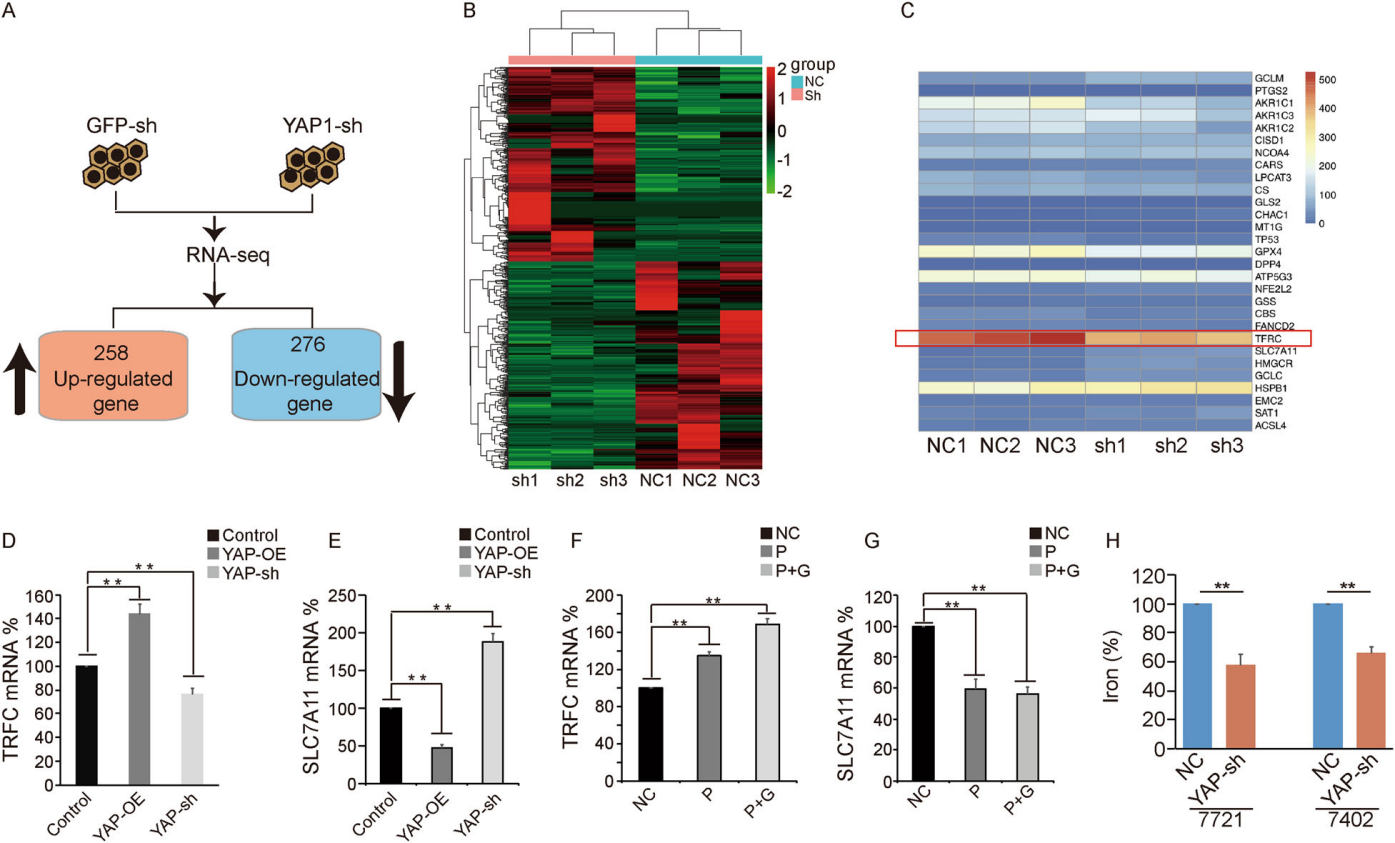
YAP regulates iron metabolism by targeting TFRC. **A** Results of RNA-seq analysis in control and YAP knockdown SMMC-7721 cells. **B** The heat map demonstrates gene clustering with three biological replicates. Bright green indicates underexpression, and bright red indicates overexpression. **C** Ferroptosis-related genes were selected to generate a new integrated heat map according to the RNA-seq data. **D** and **E** Transcription levels of TFRC (**D**) and SLC7A11 (**E**) were verified by qPCR in SMMC-7721 cells with YAP overexpression or knockdown. **F** and **G** Transcription levels of TFRC (**F**) and SLC7A11 (**G**) were measured by qPCR in SMMC-7721 cells treated with PUGNAc with or without GlcNAc. **H** The concentration of iron was determined in Bel-7402 and SMMC-7721 cells with YAP knockdown.

To further explore the underlying mechanism by which YAP enhances ferroptosis sensitivity, we decreased YAP expression and performed RNA sequencing (RNA-Seq) analysis to identify YAP target genes (Fig. 3A, Supplementary data 2). By analyzing the data with a high cutoff value (fold change > 2, *P* < 0.05), we identified 258 upregulated genes and 276 downregulated genes, as shown in the heat map (Fig. 3B). Furthermore, we selected ferroptosis-related genes to generate a new integrated heat map (Fig. 3C). Among these genes, TFRC attracted our attention, and we found that TFRC expression was significantly reduced when YAP was knocked down (Fig. 3C, Supplementary data 3). Further, we conducted qPCR to verify the RNA-seq results, and we found that knockdown of YAP led to a significant reduction in the TFRC mRNA level (Fig. 3D). Similarly, by treating cells with PUGNAc with or without GlcNAc, we determined that induction of O-GlcNAcylation also increased the TFRC mRNA level (Fig. 3F). In addition, another key ferroptosis regulator, SLC7A11, was also regulated by YAP, according to the RNA-seq data (Fig. 3C). In contrast, knockdown of YAP led to an increase in SLC7A11 mRNA expression (Fig. 3E), and treatment with PUGNAc with or without GlcNAc reduced the SLC7A11 mRNA level (Fig. 3G). TFRC (the TF receptor), as a key ferroptosis regulator, can interact with TF, which is loaded with iron, to regulate the total iron level in cells. Thus, we assessed the iron level when YAP was knocked down. As expected, decreased YAP expression obviously reduced the iron level (Fig. 3H). Taken together, these results indicated that YAP can regulate the iron level by targeting TFRC mRNA expression.

### YAP enhances ferroptosis sensitivity by regulating TFRC expression

**Fig. 4:**
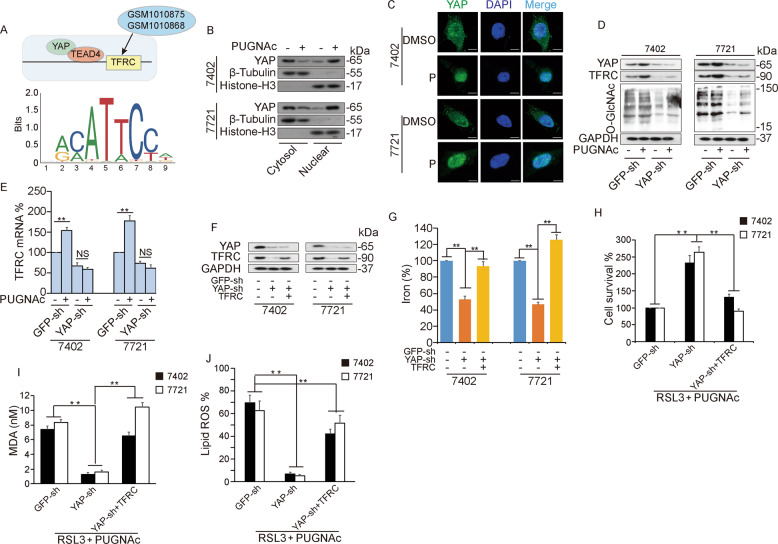
YAP enhances ferroptosis sensitivity by targeting TFRC. **A** Bioinformatics analysis of ChIP-seq data acquired from public datasets GSM1010875 and GSM1010868. **B** Cytosolic and nuclear fractionation experiments in Bel-7402 and SMMC-7721 cells with or without PUGNAc treatment. YAP was detected using WB. **C** The subcellular localization of YAP was shown using an IF assay in Bel-7402 and SMMC-7721 cells with or without PUGNAc treatment. **D** and **E** Expression of YAP, expression of TFRC, and global O-GlcNAcylation were detected by WB (**D**), and the transcription levels of TFRC were measured by qPCR (**E**) in PUGNAc-treated control and YAP knockdown Bel-7402 and SMMC-7721 cells. **F** and **G** The protein expression levels of YAP and TFRC (**F**) and the iron level (**G**) were determined in Bel-7402 and SMMC-7721 cells with simultaneous knockdown of YAP and overexpression of TFRC. **H**–**J** Ferroptotic events were evaluated in control Bel-7402 and SMMC-7721 cells and Bel-7402 and SMMC-7721 cells with simultaneous YAP knockdown and TFRC overexpression after RSL3 and PUGNAc pretreatment: cell death (**H**), lipid ROS production (**I**), and MDA production (**J**).

Further, we conducted a bioinformatics analysis using ChIP-seq datasets GSM1010875 and GSM1010868 and found that TFRC is a valid target of YAP (Fig. 4A), which confirmed our RNA-seq findings. YAP, which functions as a transcriptional cofactor, can translocate from the cytoplasm to the nucleus. O-GlcNAcylation of YAP, which can be induced by PUGNAc, increased YAP nuclear localization (Fig. 4B, C), which was reported in our previous study. Moreover, PUGNAc-induced global O-GlcNAcylation promoted YAP expression, thereby leading to increased TFRC expression at the protein and mRNA levels (Fig. 4D, E). However, this effect was abolished after YAP was inhibited (Fig. 4D, E). To explore the potential role of TFRC in YAP-enhanced ferroptosis sensitivity, we knocked down YAP expression with or without simultaneous TFRC overexpression (Fig. 4F). As the results indicated, iron accumulation was obviously inhibited by YAP knockdown and was rescued by TFRC overexpression (Fig. 4G). Subsequently, we assessed typical ferroptotic events, including cell death and the production of lipid ROS and MDA. Simultaneous TFRC overexpression significantly reduced YAP-enhanced ferroptosis sensitivity, as assessed by cell viability assays (Fig. 4H), along with the production of MDA (Fig. 4I) and lipid ROS (Fig. 4J). Moreover, we explored the potential roles of SLC7A11 in YAP-enhanced ferroptosis sensitivity, since SLC7A11 is regulated by YAP and O-GlcNAcylation. By knocking down YAP and simultaneously overexpressing SLC7A11, we found that SLC7A11 could not rescue YAP-induced ferroptosis (Supplementary Fig. 3A–D). Taken together, these results suggested that YAP enhances ferroptosis sensitivity by regulating TFRC expression.

### YAP regulates TFRC expression by binding directly to its promoter region

**Fig. 5:**
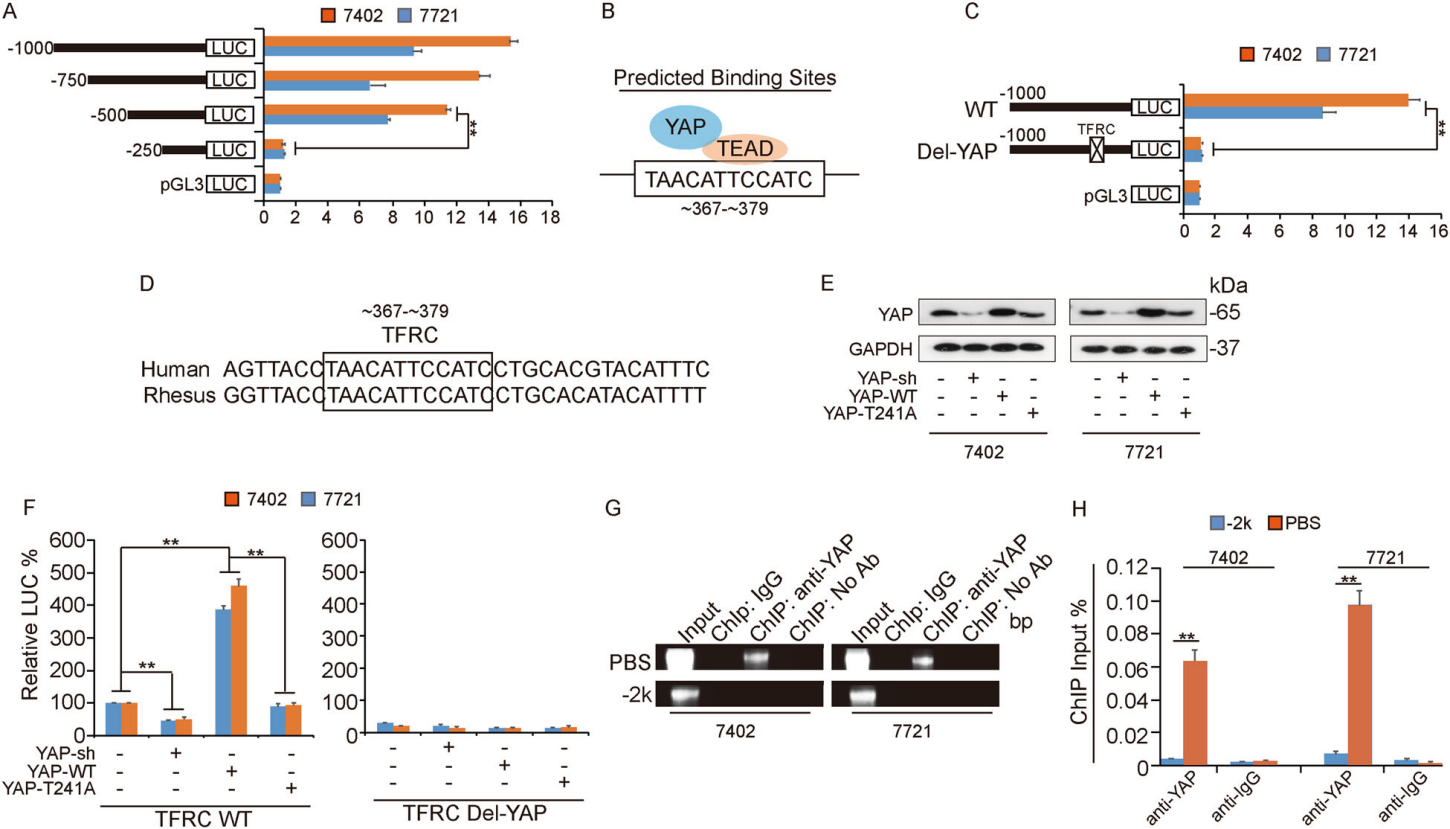
YAP regulates TFRC expression by binding to its promoter region. **A** Luciferase activity was measured with a dual-luciferase system using the indicated truncations of the TFRC promoter in Bel-7402 and SMMC-7721 cells. **B** Predicted binding site (PBS) of TFRC according to bioinformatics analysis. **C** The PBS was deleted, and the corresponding construct was referred to as Del-YAP. Luciferase activity was measured in Bel-7402 and SMMC-7721 cells. **D** Regions of PBS are relatively conserved between humans and rhesus macaques. **E** and **F** YAP expression was detected by WB (**E**), and TFRC promoter activity was measured with the dual-luciferase system (**F**) in Bel-7402 and SMMC-7721 cells with simultaneous knockdown of YAP and overexpression of YAP-WT or YAP-T241A. **G** and **H** Chromatin was immunoprecipitated using an anti-YAP antibody or negative control anti-IgG antibody (**G**), and qPCR (H) was then conducted in Bel-7402 and SMMC-7721 cells.

We identified TFRC as a valid target of YAP, according to the ChIP-seq datasets (Fig. 4A). Thus, we generated the indicated reporters that contained TFRC promoter mutants with truncations at 250 intervals from the translation start site (TSS) (Fig. 5A). When the −500 to −250 nt region was deleted, the basal promoter activity of TFRC was diminished to a level similar to that of the pGL3 control vector (Fig. 5A), suggesting that this region might be responsible for YAP binding. Next, we conducted bioinformatics analysis to predict the putative binding site (PBS) (Fig. 5B). More intriguingly, the PBS was located in the −500 to −250 nt region of the TFRC promoter. Then, a TFRC promoter reporter vector with precise deletion of PBS (Del-YAP) was constructed, and it was found that transfection of the Del-YAP plasmid led to total loss of TFRC promoter activity (Fig. 5C). Moreover, the PBS sequence was relatively conserved between humans and rhesus macaques (Fig. 5D). To confirm that YAP can regulate TFRC promoter activity, we transfected cells with the YAP-sh plasmid, YAP-WT plasmid, or YAP-T241A mutant plasmid (Fig. 5E). As expected, knockdown of YAP inhibited TFRC promoter activity, and overexpression of YAP-WT but not YAP-T241A significantly facilitated TFRC promoter activity (Fig. 5F). Importantly, changes in YAP expression (wild-type YAP or mutant YAP) had no effect on the activity of the TFRC promoter with deletion of the PBS (Del-YAP) (Fig. 5F). In addition, we conducted a ChIP assay to confirm the physical binding between YAP and the TFRC promoter (Fig. 5G). As the results suggested, the YAP-binding round of the PBS was confirmed, and the region 2k upstream of the TSS showed no occupancy of YAP (Fig. 5I). These results indicated that YAP can bind directly to the TFRC promoter region and regulate TFRC expression.

### O-GlcNAcylation enhanced ferroptosis sensitivity by regulating TFRC

**Fig. 6:**
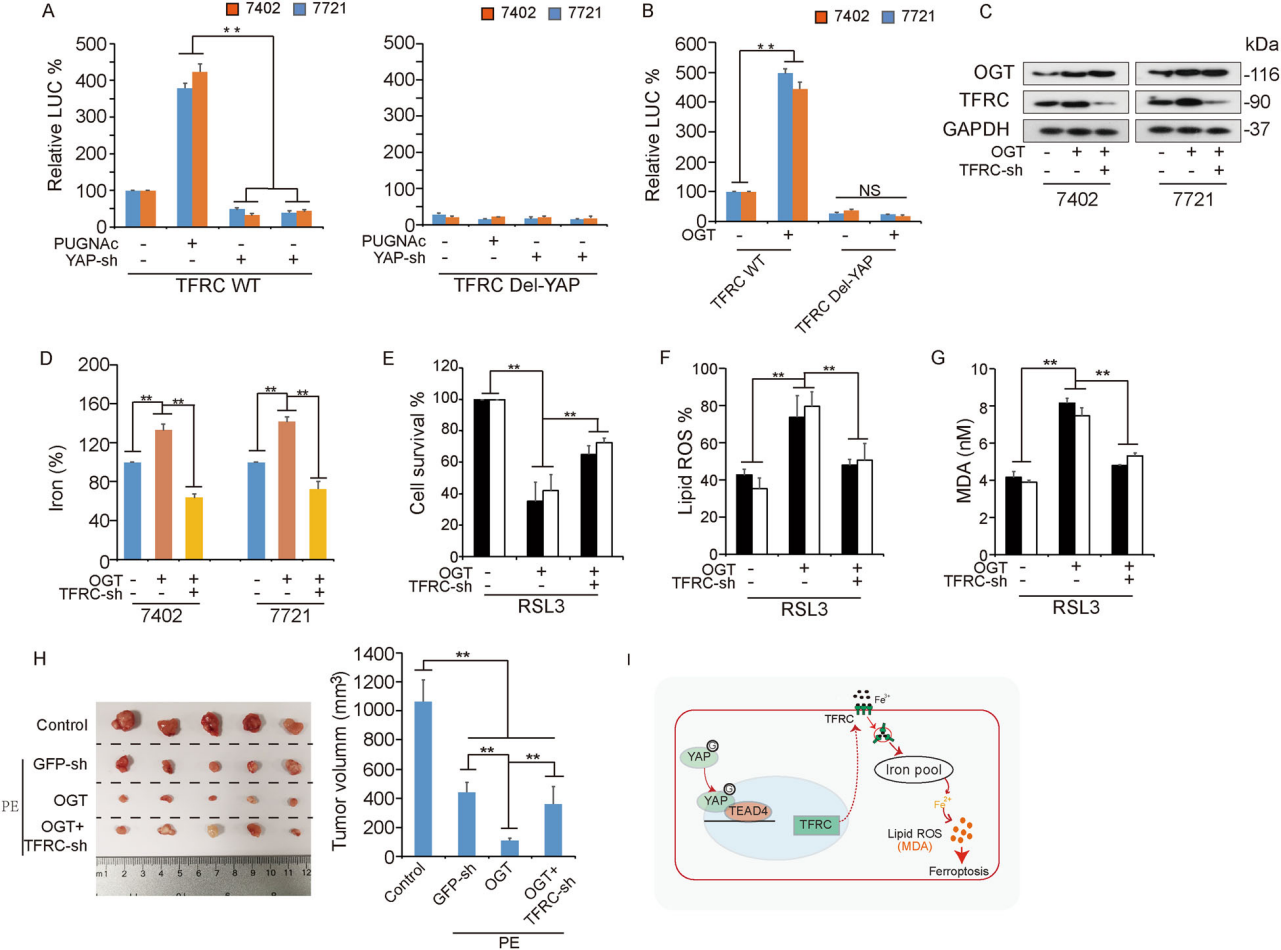
O-GlcNAcylation enhances ferroptosis sensitivity by regulating TFRC. **A** The luciferase activity of reporters containing wild-type TFRC and Del-YAP was measured in Bel-7402 and SMMC-7721 cells with or without YAP knockdown and PUGNAc treatment. **B** The luciferase activity of the reporters containing TFRC and Del-YAP was measured in Bel-7402 and SMMC-7721 cells with OGT overexpression. **C**–**G** OGT was overexpressed in Bel-7402 and SMMC-7721 cells with simultaneous TFRC knockdown (**C**). The iron concentration in cells treated as indicated was determined (**D**). Ferroptotic events in cells treated as indicated were evaluated: cell death (**E**), lipid ROS production (**F**), and MDA production (**G**). **H** A xenograft assay was performed using Bel-7402 cells treated as indicated with or without piperazine erastin treatment. **I** Schematic abstract of the possible mechanism by which O-GlcNAcylation promotes ferroptosis sensitivity via the YAP/TFRC pathway in HCC cells.

Next, we found by a luciferase assay that PUGNAc-stimulated O-GlcNAcylation enhanced TFRC promoter activity (Fig. 6A). The PUGNAc-stimulated increase in the TFRC transcription level was abolished by YAP inhibition. PUGNAc treatment had no effect on the luciferase activity of the TFRC promoter with deletion of the TFRC motif (Del‐TFRC). Furthermore, OGT-induced O-GlcNAcylation confirmed our finding (Fig. 6B). Subsequently, we overexpressed OGT to upregulate global O-GlcNAcylation and simultaneously knocked down TFRC (Fig. 6C). We found that the OGT overexpression-mediated reduction in iron levels was reversed by simultaneous inhibition of TFRC (Fig. 6D). Furthermore, OGT-induced ferroptosis sensitivity was reversed by simultaneous inhibition of TFRC (Fig. 6E–G). Additionally, treatment with piperazine erastin (a form of erastin with increased stability in vivo) reduced the volume of xenografts derived from OGT-overexpressing cells compared to xenografts derived from GFP-sh cells (Fig. 6H). Taken together, these results indicated that O-GlcNAcylation enhances ferroptosis sensitivity by regulating TFRC.

## Discussion

Ferroptosis is a novel form of cell death characterized by iron-dependent accumulation of peroxidated PUFA-containing phospholipids. Despite decades of advances in cancer treatment, eliminating cancer cells while leaving normal cells unharmed remains challenging. Recently, ferroptosis has emerged as a tumor suppressor, providing alternative strategies for cancer treatment. For instance, the classical tumor suppressor p53 facilitates ferroptosis by inhibiting cysteine intake via regulation of SLC7A11 expression^1,23,24^. Chen et al. proved that ATF4 suppression constitutes a valid therapeutic target for cancer by sensitizing tumor cells to ferroptosis^25^. In addition, Luo et al. found that miR-137 might contribute to ferroptosis by suppressing glutaminolysis in melanoma and might be a potential therapeutic target^26^. In the present study, we first suggested that YAP O-GlcNAcylation can enhance ferroptosis sensitivity in HCC cells by regulating TFRC expression and can be regarded as a therapeutic approach. TFRC, which is a membrane-located protein that is a receptor for iron-loaded TF, plays pivotal roles in mediating ferroptosis^27^. Targeting TFRC expression to reduce the amount of iron available to tumors is frequently regarded as a therapeutic strategy in various cancer cells^28,29^.

To adapt to the rapid growth of tumors, cells increase their iron uptake, which also endows them with enhanced sensitivity to iron-dependent cell death, referred to as ferroptosis. Iron is a redox-active metal that can participate in the formation of free radicals and the production of lipid peroxides, which is mediated by the Fenton reaction^30^. Therefore, regulating the levels of iron can increase vulnerability to ferroptosis. Numerous proteins involved in iron metabolism have been implied to regulate ferroptosis. Suppression of NFS1, which is an iron–sulfur cluster biosynthetic enzyme, promotes TFRC expression and sensitizes cells to ferroptosis^31^. NCOA4, a cargo receptor that mediates autophagic degradation of ferritin (also called ferritinophagy), contributes to ferroptosis by controlling ferritin degradation and iron release^32^. Moreover, Sun et al. and Yuan et al. reported that HSPB1 and CISD1, respectively, can influence ferroptosis sensitivity by targeting iron metabolism^33,34^.

More importantly, ferroptosis and O-GlcNAcylation closely interact with cellular metabolism. For instance, Gao et al. reported that the degradation of glutamine, also known as glutaminolysis, is required for cysteine deprivation-induced ferroptosis^13^. They found that as a decomposition product of glutaminolysis, glutamate was subsequently converted into α-ketoglutarate (α-KG), which participates in the tricarboxylic acid (TCA) cycle and lipid ROS production. In addition, glutamine directly fuels the HBP pathway to control global O-GlcNAcylation^35^. Remarkably, glutamine is connected to both ferroptosis and O-GlcNAcylation by its dual contributions to the TCA cycle and HBP pathway. This phenomenon inspired us to hypothesize that there might be some connection between O-GlcNAcylation and ferroptosis. Furthermore, YAP function relies on O-GlcNAcylation, and YAP increases sensitivity to ferroptosis, according to Wu et al.’s study^20^, facilitating the validation of our hypothesis.

Further, another study reported that the O-GlcNAcylation-dependent upregulation of HO1 can induce ferrous iron release to promote the induction of oxidative stress. HO1 activity substantially affects iron homeostasis in ammonia-exposed astrocytes^36^. Generally, HO1 protein expression can be stimulated by O-GlcNAcylation, which is consistent with our finding.

In summary, the present study explored the underlying molecular mechanism to reveal the pivotal roles of YAP O-GlcNAcylation in enhancing ferroptosis sensitivity. O-GlcNAcylation significantly enhanced YAP transcriptional activity, thereby leading to an increase in TFRC expression. TFRC overexpression enhanced cellular iron absorption, which enhanced ferroptosis sensitivity. Inducing ferroptosis under high O-GlcNAcylation levels might be a promising therapeutic strategy for HCC.

## Materials and methods

### Cell culture and vectors

The liver cancer cell lines Bel-7402 and SMMC-7721 were obtained from the Cell Bank of the Chinese Academy of Sciences (Shanghai, China). The HEK293T cell line was obtained from the American Type Culture Collection (Manassas, VA, USA). Cells were cultured in Dulbecco’s-modified Eagle’s medium (DMEM) supplemented with 10% fetal bovine serum and maintained at 37 °C in 5% CO_2_. For induction of ferroptosis and O-GlcNAcylation, cells were treated with PUGNAc (25 µM, Sigma, St. Louis, MO, USA) and RSL3 (2 µM, Sigma), respectively. For inhibition of ferroptosis, apoptosis, and necrosis, cells were treated with ferrostatin-1 (2 µM, Sigma), ZVAD-FMK (6 µM, Selleck), and necrosulfonamide (1 µM, Selleck), respectively. The YAP-WT, YAP-sh, OGT-OE, and OGT-sh plasmids were obtained in our previous studies^19^. The TFRC-sh, TFRC-OE, SLC7A11-sh, and SLC7A11-OE plasmids were purchased from GeneChem (Shanghai, China). The plasmids with truncated TFRC promoter regions that were cloned into pGL3 were purchased from Sangon Biotech (Shanghai, China).

### Western blotting (WB)

WB was conducted according to conventional protocols. Briefly, cells treated as indicated were harvested and lysed using RIPA buffer (Beyotime, China). Proteins were extracted and transferred to nitrocellulose membranes via SDS–PAGE. Then, membranes were blocked with 5% skimmed milk for 1 h at room temperature prior to incubation with primary antibodies overnight at 4 °C. The primary antibodies used were anti-O-GlcNAc (Abcam, #ab2735), anti-OGT (Abcam, #ab184198 or #ab177941), anti-YAP (Abcam, #ab52771), anti-β-Tubulin (CST, #2128), anti-Histone-H3 (Santa Cruz, #sc-10809), anti-TFRC (Abcam, #ab84036), anti-SLC7A11 (Abcam, #175186), and anti-GAPDH (CST, #5176). For nuclear and cytosolic separation, nuclear extraction was conducted according to the manufacturer’s protocols for the Nuclear Extraction Kit (Active Motif, USA). Membranes were incubated with HRP-linked secondary antibodies [anti-rabbit (CST, #7074) or anti-mouse (CST, #7076)] for 1 h at room temperature. Signals were detected using Pierce™ ECL Western Blotting Substrate (Thermo Scientific, USA).

### Cell proliferation and colony formation assays

Cell proliferation and colony formation assays were performed as described previously. Briefly, for the cell proliferation assay, cells treated as indicated were seeded into a 96-well plate at a density of 3000 cells per well. Then, the cells were incubated with CCK-8 reagent (Beyotime, China) for 1 h. The absorbance was measured at 450 nm. For the colony formation assay, cells were seeded into six-well plates at a density of 5000 cells per well in DMEM containing 0.3% agarose. After incubation for 2 weeks, colonies were counted.

### Enzymatic labeling of O-GlcNAc sites

The immunoprecipitated YAP (WT or T241A) with protein A/G-Sepharose were incubated with reaction buffer (20 mM HEPES, 50 mM NaCl, 1 µM PuGNAc, and 5 mM MnCl_2_ with protease and phosphatase inhibitors). Then 2 µl of Gal-T1Y289L (Invitrogen, Carlsbad, CA, USA) and 2 µl of 0.5 mM UDP-GalNAz (Invitrogen) were added. The mix was incubated overnight at 4 °C. After washing twice, the samples were reacted with biotin alkyne (Invitrogen) or tetramethyl-6-carboxyrhodamine (TAMRA) alkyne (Invitrogen) and finally detected by WB using HRP HRP-labeled Streptavidin (Beyotime, #A0303) or antibodies against TAMRA (Invitrogen, #A6397)^19^.

### Lipid ROS detection

Lipid ROS were assessed using C11-BODIPY dye (Thermo Scientific, USA) according to the manufacturer’s protocol. Briefly, cells treated as indicated were collected and incubated with DMEM containing C11-BODIPY at a final concentration of 5 µM for 20 min at 37 °C. Then, the cells were washed twice with PBS to remove residual C11-BODIPY. Then, the filtered cell suspension was subjected to flow cytometric analysis (BD Biosciences, USA).

### Malondialdehyde (MDA) detection

MDA concentrations were measured with a lipid peroxidation assay kit (Abcam, #ab118970) according to the manufacturer’s protocol. Briefly, cells treated as indicated were lysed using lysis buffer, and the supernatant was collected. Thiobarbituric acid (TBA) solution was added to the samples, and the mixture was incubated at 95 °C for 1 h. Then, the mixture was cooled to room temperature and added to a 96-well microplate. The absorbance at 532 nm was measured immediately using a microplate reader (Biotek).

### Immunofluorescence (IF) assays

IF assays were conducted according to conventional protocols. Briefly, cells treated as indicated were washed twice with PBS and fixed with 4% paraformaldehyde (PFA). The primary antibody used was anti-YAP (Abcam, #52771). After incubation at 4 °C overnight, the cells were washed with PBS and incubated with Alexa Fluor^®^-488 fluorophore-conjugated secondary antibodies (CST, #4408, #4412) at room temperature and protected from light. The slides were washed with PBS and stained with ProLong^®^ Gold anti-fade reagent with DAPI (Molecular Probes, USA). Subsequently, the slides were visualized with an LSM 800 confocal microscope (Carl Zeiss, Germany).

### Coimmunoprecipitation (co-IP)

Co-IP was conducted according to conventional protocols. Briefly, cells were harvested and lysed using RIPA lysis buffer. Proteins were extracted and mixed with protein A/G-Sepharose beads (Novex, Norway) and the indicated antibodies at 4 °C overnight. The beads were washed twice with RIPA buffer and resuspended in SDS–PAGE loading buffer prior to WB. The antibody used was anti-YAP (Abcam, #ab52771).

### Chromatin immunoprecipitation (ChIP) assay

The ChIP assay was performed with a ChIP-IT express kit according to the manufacturer’s protocol (Active Motif, CA). Cells treated as indicated were fixed with 1% formaldehyde for 8 min at room temperature. Crosslinking was terminated with glycine at a final concentration of 0.125 M. Sonication was conducted for four cycles (25 s on and 25 s off) with a Qsonica sonicator. Subsequently, the supernatant was incubated with protein A/G-Sepharose beads and antibodies at 4 °C overnight. Protein–antibody–DNA complexes were washed, crosslinking was reversed, and DNA was then purified using a DNA Purification Kit (Beyotime, China). The purified DNA was subjected to qPCR analysis. The antibody used was anti-YAP (Abcam, #ab52771). The primers used for ChIP are listed in Supplementary Table 1.

### Iron detection

Iron detection was conducted using an Iron Assay Kit (Abcam) according to the manufacturer’s protocol to assess the total iron concentration. Generally, cells treated as indicated were harvested and lysed using iron assay buffer. An iron reducer was added to the supernatant and incubated at room temperature for 30 min in the dark. Subsequently, the iron probe was added, and the mixture was incubated for 1 h at room temperature. The absorbance at 593 nm was measured immediately using a microplate reader.

### Dual-Luciferase reporter assay

Luciferase reporter vectors were transfected into the Bel-7402 and SMMC-7721 cell lines along with the Renilla luciferase expression plasmid. Cells were incubated for 48 h and lysed with passive lysis buffer according to the instructions of a dual-luciferase system (Promega, WI). Signals were measured immediately using a microplate reader.

### Quantitative RT-PCR (qPCR)

Total RNA was extracted according to conventional protocols using TRIzol (Invitrogen, USA). cDNA was synthesized with a PrimeScript™ RT Reagent Kit (Takara, China). qPCR was carried out using TB Green^®^ Premix Ex Taq™ II (Takara, China). mRNA expression levels were normalized to GAPDH expression levels. The primers used were as follows: TFRC, F: GCTCGGCAAGTAGATGGCGATAAC; R: ATTGTCAATGTCCCAAACGTCACC. SLC7A11, F: AGCCTGTTGTGTCCACCATCTCC; R: GTCAGAGTGATGACGAAGCCAATC. GAPDH, F: ATCATCCCTGCCTCTACTGG; R: GTCAGGTCCACCACTGACAC.

### Mouse experiments

Five-week-old nude mice were purchased from Bikai Laboratory Animal Corp. (Bikai, China). Bel-7402 cells treated as indicated were subcutaneously injected into mice at a density of 5 × 10^6^. After xenografts were formed, piperazine erastin, which was purchased from MedChemExpress, was subcutaneously injected into the mice once a day. The tumors were measured 14 days after piperazine erastin injection, and tumor volumes were calculated with the formula 0.5 × *L* × *W*^2^, where *L* represents the length and *W* represents the width. The animal experiments were conducted under the institutional guidelines of Shanghai Children’s Medical Center.

### RNA-seq analysis

RNA-seq analysis was performed at Genminix Informatics Co., Ltd. (Shanghai, China). In brief, total RNA was extracted from SMMC-7721 cells with or without YAP knockdown. After quality analysis in an Agilent 4200 Bioanalyzer, samples were submitted for cDNA library construction. Then, the prepared libraries were sequenced on the Illumina HiSeq 2000 platform. Differential expression analysis was performed using the DESeq R package. Genes with an adjusted *p* value < 0.05 were considered differentially expressed.

### Statistical analysis

The significance of differences was tested using Student’s *t*-test or one-way ANOVA. *p* < 0.05 indicates a statistically significant difference. Analysis was performed using SPSS v22.0 (IBM, USA).

## Supplementary information

supplementary figure legends

revised-supplementary figure1

revised-supplementary figure2

revised-supplementary figure3

supplementary data1. Primers used for PCR

supplementary data 2. differential expression genes after YAP knocked down

supplementary data 3. Ferroptosis related genes expression after YAP knocked down

## References

[1] Dixon SJ (2012). Ferroptosis: an iron-dependent form of nonapoptotic cell death. Cell.

[2] Pasparakis M, Vandenabeele P (2015). Necroptosis and its role in inflammation. Nature.

[3] Vanden Berghe T, Hassannia B, Vandenabeele P (2016). An outline of necrosome triggers. Cell Mol. Life Sci..

[4] Dolma S, Lessnick SL, Hahn WC, Stockwell BR (2003). Identification of genotype-selective antitumor agents using synthetic lethal chemical screening in engineered human tumor cells. Cancer Cell.

[5] Tang D, Kroemer G (2020). Ferroptosis. Curr. Biol..

[6] Yang WS (2014). Regulation of ferroptotic cancer cell death by GPX4. Cell.

[7] Doll S (2017). ACSL4 dictates ferroptosis sensitivity by shaping cellular lipid composition. Nat. Chem. Biol..

[8] Chen YC (2020). Reactivity-based probe of the Iron(II)-dependent interactome identifies new cellular modulators of ferroptosis. J. Am. Chem. Soc..

[9] Xu, X., Chen, Y., Zhang, Y., Yao, Y. & Ji, P. Highly stable and biocompatible hyaluronic acid-rehabilitated nanoscale MOF-Fe(2+) induced ferroptosis in breast cancer cells. *J. Mater. Chem. B* (2020). 10.1039/d0tb01616k 32944722

[10] Hong, X. et al. The lipogenic regulator SREBF2 induces Transferrin in circulating melanoma cells and suppresses ferroptosis. *Cancer Discov.* **11**, 678–695 (2021). 10.1158/2159-8290.CD-19-1500 PMC7933049 33203734

[11] Tang, L. J. et al. Ubiquitin-specific protease 7 promotes ferroptosis via activation of the p53/TfR1 pathway in the rat hearts after ischemia/reperfusion. *Free Radic. Biol. Med.* **162**, 339–352 (2021). 10.1016/j.freeradbiomed.2020.10.307 33157209

[12] Yang WS, Stockwell BR (2008). Synthetic lethal screening identifies compounds activating iron-dependent, nonapoptotic cell death in oncogenic-RAS-harboring cancer cells. Chem. Biol..

[13] Gao M, Monian P, Quadri N, Ramasamy R, Jiang X (2015). Glutaminolysis and transferrin regulate ferroptosis. Mol. Cell.

[14] Hassannia B, Vandenabeele P, Vanden Berghe T (2019). Targeting ferroptosis to iron out cancer. Cancer Cell.

[15] Chatham, J. C., Zhang, J. & Wende, A. R. Role of O-linked N-acetylglucosamine protein modification in cellular (patho)physiology. *Physiol. Rev.* **101**, 427–493 (2021). 10.1152/physrev.00043.2019 PMC8428922 32730113

[16] Hu CM (2019). High glucose triggers nucleotide imbalance through O-GlcNAcylation of key enzymes and induces KRAS mutation in pancreatic cells. Cell Metab..

[17] Duan F (2018). O-GlcNAcylation of RACK1 promotes hepatocellular carcinogenesis. J. Hepatol..

[18] Yang X, Qian K (2017). Protein O-GlcNAcylation: emerging mechanisms and functions. Nat. Rev. Mol. Cell Biol..

[19] Zhang X (2017). The essential role of YAP O-GlcNAcylation in high-glucose-stimulated liver tumorigenesis. Nat. Commun..

[20] Wu J (2019). Intercellular interaction dictates cancer cell ferroptosis via NF2-YAP signalling. Nature.

[21] Kim J, DeBerardinis RJ (2019). Mechanisms and implications of metabolic heterogeneity in cancer. Cell Metab..

[22] Peng C (2017). Regulation of the Hippo-YAP pathway by glucose sensor O-GlcNAcylation. Mol. Cell.

[23] Yagoda N (2007). RAS-RAF-MEK-dependent oxidative cell death involving voltage-dependent anion channels. Nature.

[24] Jiang L (2015). Ferroptosis as a p53-mediated activity during tumour suppression. Nature.

[25] Chen D (2017). ATF4 promotes angiogenesis and neuronal cell death and confers ferroptosis in a xCT-dependent manner. Oncogene.

[26] Luo M (2018). miR-137 regulates ferroptosis by targeting glutamine transporter SLC1A5 in melanoma. Cell Death Differ..

[27] Bogdan AR, Miyazawa M, Hashimoto K, Tsuji Y (2016). Regulators of iron homeostasis: new players in metabolism, cell death, and disease. Trends Biochem. Sci..

[28] Daniels TR (2012). The transferrin receptor and the targeted delivery of therapeutic agents against cancer. Biochim. Biophys. Acta.

[29] Horonchik L, Wessling-Resnick M (2008). The small-molecule iron transport inhibitor ferristatin/NSC306711 promotes degradation of the transferrin receptor. Chem. Biol..

[30] Melin V, Henriquez A, Freer J, Contreras D (2015). Reactivity of catecholamine-driven Fenton reaction and its relationships with iron(III) speciation. Redox Rep..

[31] Alvarez SW (2017). NFS1 undergoes positive selection in lung tumours and protects cells from ferroptosis. Nature.

[32] Gao M (2016). Ferroptosis is an autophagic cell death process. Cell Res..

[33] Sun X (2015). HSPB1 as a novel regulator of ferroptotic cancer cell death. Oncogene.

[34] Yuan H, Li X, Zhang X, Kang R, Tang D (2016). CISD1 inhibits ferroptosis by protection against mitochondrial lipid peroxidation. Biochem. Biophys. Res. Commun..

[35] Swamy M (2016). Glucose and glutamine fuel protein O-GlcNAcylation to control T cell self-renewal and malignancy. Nat. Immunol..

[36] Gorg B (2019). O-GlcNAcylation-dependent upregulation of HO1 triggers ammonia-induced oxidative stress and senescence in hepatic encephalopathy. J. Hepatol..

