# Supplementary material for: O-GlcNAcylation enhances sensitivity to RSL3-induced ferroptosis via the YAP/TFRC pathway in liver cancer
Source: Cell Death Discov. 2021 Apr 16;7:83. doi: 10.1038/s41420-021-00468-2 (PMC8052337; doi:10.1038/s41420-021-00468-2)
Supplement: Supplementary file 1 — supplementary figure legends [file 41420_2021_468_MOESM1_ESM.docx]

Supplementary Figure Legends

Supplementary Figure 1: A-B) cell viability was measured by MTT assay in 7402 cell (A) and 7721 cell (B) with the indicated treatment. C) global O-GlcNAcylation and OGT expression were detected by WB after OGT knocked down with or without RSL3 treatment simultaneously. D-F) Ferroptotic events including cell viability (D), lipid ROS (E) and MDA (F) were measured in control and 7402 or 7721 cells under the indicated treatments. G) cell viability was detected by PI-staining under the indicated treatment in 7402 or 7721 cells.

Supplementary Figure 2: A) Interaction between O-GlcNAc and YAP by using anti-O-GlcNAc antibodies in 7402 and 7721 cells. B) YAP O-GlcNAcylation was measured via HRP-labelled Streptavidin (left) and anti-TAMRA antibodies (right) in 7402 and 7721 cells. (C) Enzymatic labelling of O-GlcNAc in WT and T241A YAP-FLAG proteins as analysed by anti-TAMRA antibodies and HRP-labelled Streptavidin in 7402 and 7721 cells.

Figure 3: A) YAP, SLC7A11 protein expression levels were detected in 7402 and 7721 cells with YAP knocked down and simultaneously overexpression of SLC7A11. (B-D) Ferroptotic events were measured in control and 7402 or 7721 cells with YAP knocked down and simultaneously SLC7A11 overexpression after RSL3 and PUGNAc pretreatment, including cell viability (B), lipid ROS (C) and MDA (D)
