# Supplementary figures and images for: O-GlcNAcylation enhances sensitivity to RSL3-induced ferroptosis via the YAP/TFRC pathway in liver cancer
Source: Cell Death Discov. 2021 Apr 16;7:83. doi: 10.1038/s41420-021-00468-2 (PMC8052337; doi:10.1038/s41420-021-00468-2)

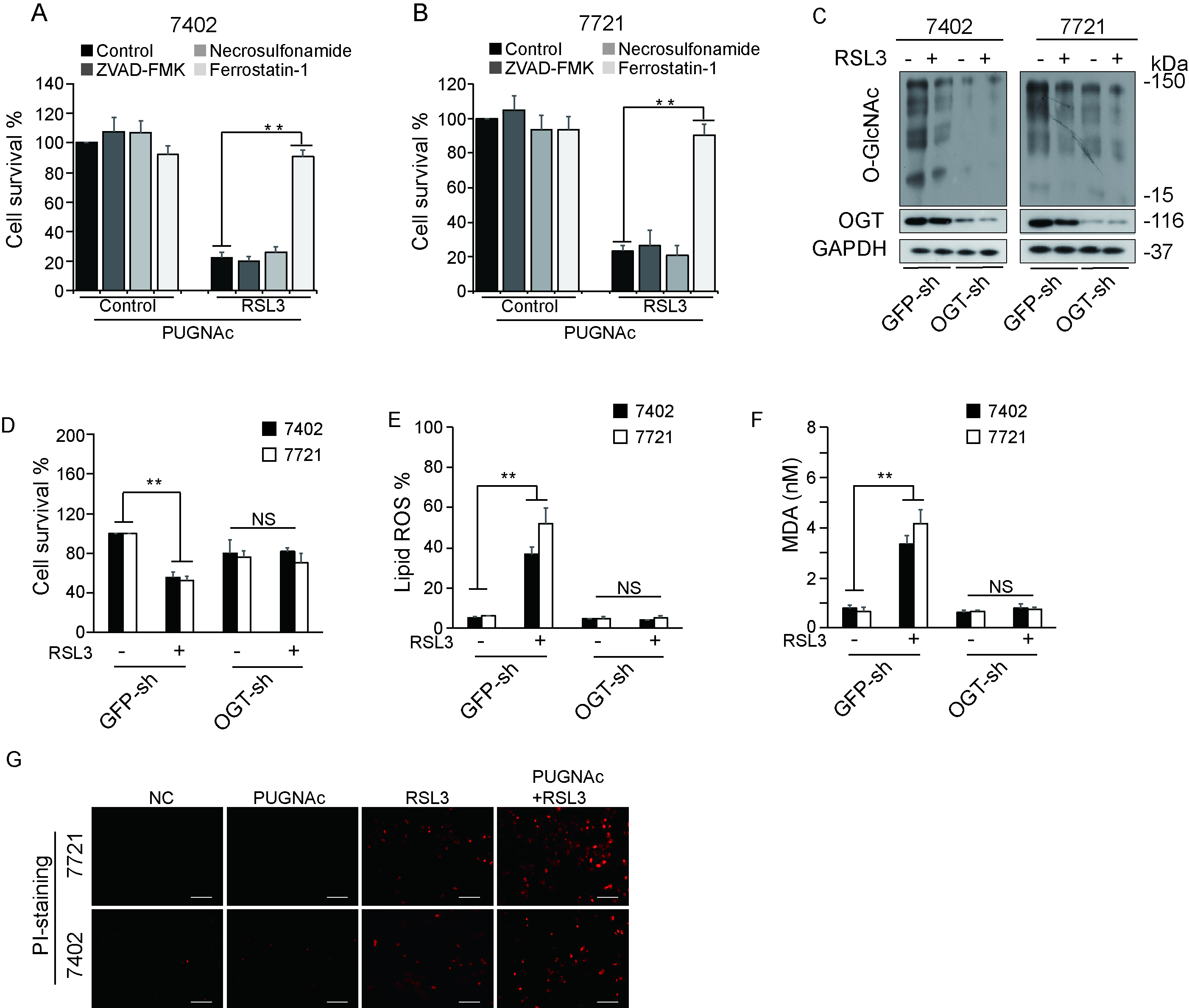

Supplement: Supplementary file 2 — revised-supplementary figure1 [file 41420_2021_468_MOESM2_ESM.tif]

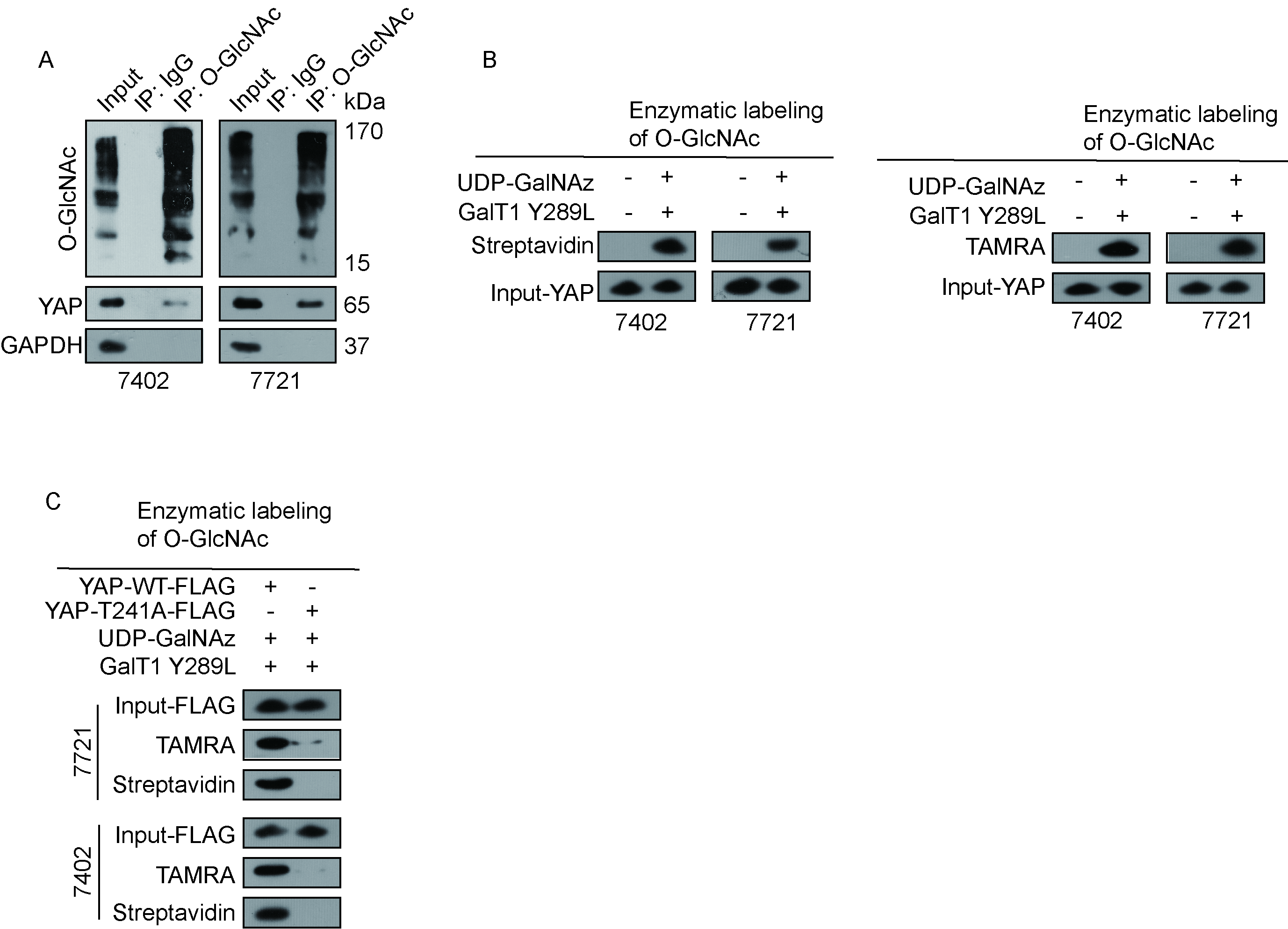

Supplement: Supplementary file 3 — revised-supplementary figure2 [file 41420_2021_468_MOESM3_ESM.tif]

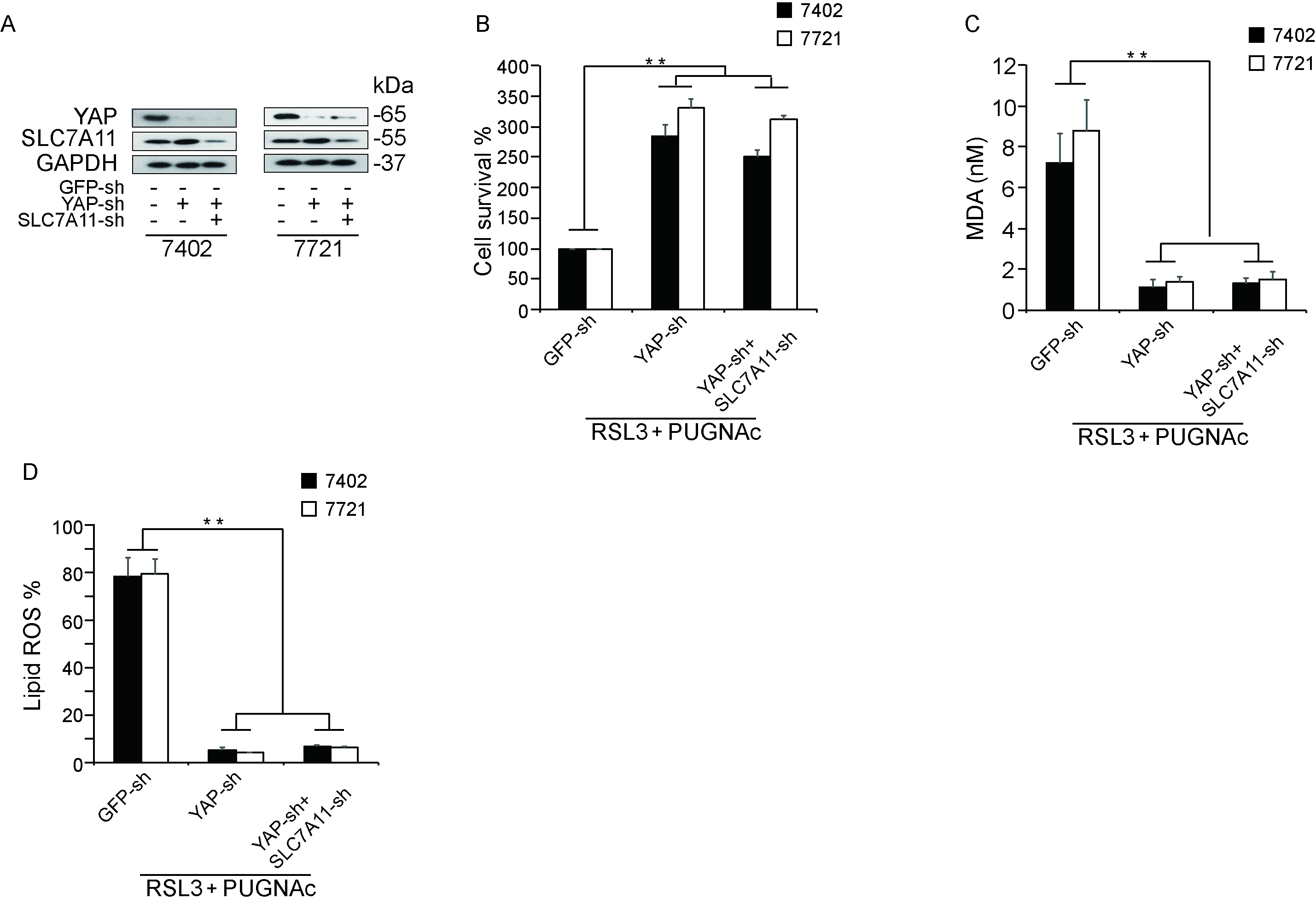

Supplement: Supplementary file 4 — revised-supplementary figure3 [file 41420_2021_468_MOESM4_ESM.tif]
